# Synapse and dendrite deficits induced by mutations in the X-linked intellectual disability gene Il1rapl1

**DOI:** 10.1186/2193-1801-4-S1-P29

**Published:** 2015-06-12

**Authors:** Caterina Montani, Mariana Ramos-Brossier, Mariana Ramos-Brossier, Carlo Sala

**Affiliations:** CNR Neuroscience Institute, Italy; Institut Cochin, INSERM, Université Paris Descartes, France

**Keywords:** Synapse, intellectual disability, autism spectrum disorder

Synapse and dendrite deficits induced by mutations in the X-linked intellectual disability gene Il1rapl1 Caterina Montani, Mariana Ramos-Brossier, Pierre Billuart, Carlo Sala Mutations and deletions of Interleukin-1 receptor accessory protein like 1 (IL1RAPL1) gene, localized on X chromosome, are associated to intellectual disability (ID) and autism spectrum disorder (ASD). IL1RAPL1 protein is localized at the postsynaptic compartment of excitatory synapses and plays a role in synapse formation and stabilization. Our project was to characterize IL1RAPL1 mutants identified in patients with ID and ASD and to perform a behavioral and neuronal morphology analysis on IL1RAPL1 KO mice. Specifically, we studied the function of three novel mutations of IL1RAPL1 gene in patients presenting ID. We found that two of the studied mutants lead to a partial loss of function of IL1RAPL1 and we pointed out the important function of the extracellular domain for the trans-synaptic PTPδ/IL1RAPL1 interaction in synaptogenesis. We also characterized the role of IL1RAPL1 wild type and mutants in regulating dendrite morphology using in vitro neuronal cultures and IL1RAPL1 KO mice. We identified, associated to hippocampal cognitive impairment an increased number of dendrite branching points in CA1 and CA2 hippocampal neurons of IL1RAPL1 KO mice. In transfected hippocampal neurons the overexpression of full length IL1RAPL1 and mutants lacking part of C-terminal domains leads to a simplification of neuronal arborisation. This effect is abolished when we overexpressed mutants lacking part of N-terminal domains. Our results indicate the importance of IL1RAPL1 extracellular domains not only in synaptogenesis but also in dendrite development. We also concluded that for this activity PTPδ interaction is not required, suggesting that an unknown IL1RAPL1 binding partner is involved in the effect on dendrite morphology.

